# Mismatched menu: the incompatibility of adult black soldier flies as praying mantis feed

**DOI:** 10.3389/finsc.2025.1531683

**Published:** 2025-03-19

**Authors:** Patrick Klüber, Raissa Gabche

**Affiliations:** Department of Bioresources, Fraunhofer Institute for Molecular Biology and Applied Ecology, Giessen, Germany

**Keywords:** *Hermetia illucens*, praying mantis, terraristics, insect feed, insect rearing, insects as food and feed, feeding behavior

## Abstract

Praying mantises are known for their striking predatory behavior and are becoming increasingly popular with hobbyists and for scientific research. As generalist predators with a wide range of insect-based diets, it is crucial to identify suitable prey options, especially for restricted environments such as terrariums, which are limited compared to the wild. This study investigates the use of adult black soldier flies (BSF; *Hermetia illucens;* Linnaeus, 1758) as a sole food source for two mantis species, *Chlidonoptera lestoni* (Roy & Leston, 1975) and *Hierodula patellifera* (Serville, 1839), while assessing their suitability and potential challenges associated with their digestion. The BSF is widely recognized for its high nutritional value and ease of rearing, making it an attractive prey candidate for mantises. Although natural capture behavior and high feed acceptance have been observed, adult BSF seem not to be suitable as sole feed for both mantises. Our results suggest that imbalances in macronutrients, particularly the protein/fat ratio, may contribute to high mortality. The use of BSF as a mono-diet could also limit access to a variety of beneficial microorganisms that are essential for maintaining a healthy gut microbiota in mantises, thereby affecting their immunity and well-being in captivity. In addition, the possible presence of pathogenic microorganisms in the BSF could also have affected the mantises’ survival. Future studies should focus on the nutritional adjustment of BSF, as their chemical composition is strongly dependent on the feed they are reared on.

## 1 Introduction

Praying mantises (Mantodea) are fascinating insects known for their distinctive predatory behavior and unique appearance. There are approximately 2,500 species worldwide, most of which are found in tropical and subtropical regions (1, 2). However, some species are native to more temperate climates, such as *Mantis religiosa* (Mantodea: Mantidae; Linnaeus, 1758), widely distributed throughout Europe, especially in the Euro-Mediterranean region, with a variety of vegetation and climates (3, 4). Mantises naturally feed on various insects including flies, crickets, moths, beetles and cockroaches (5–7), but in rare cases it has even been observed that they consume small vertebrates such as frogs, lizards and birds (8–10). Furthermore, cannibalistic behavior is not unusual, especially during mating when food supplies can be low and the nutritional requirements of females are not met (7). Their highly predatory behavior is important for their nutrition and survival, as they are carnivorous insects and feed primarily on live and moving prey, with their reaction and responsiveness increasing under conditions of prolonged starvation (11). The carnivorous diet provides them with a rich source of protein, fat and other essential nutrients necessary for proper growth and development (12). Specialized raptorial forelegs allow for rapid movement to capture prey and are equipped with spines that enable the mantis to control and hold its prey while eating (13). The number and arrangement of these spines are specialized according to their diet and ecology. Even the mouthparts can vary in composition based on the type of prey they consume (10, 14–16). As ambush predators, they also rely on their keen eyesight and flexible neck enabling them to rotate their heads up to 180 degrees (17, 18).

Hobbyists and researchers are increasingly interested in keeping and caring for arthropods such as praying mantises for behavioral studies and natural pest control (19). Therefore, confined and controlled environments are created by using variously equipped plastic containers or terrariums to provide a habitat that mimics the natural environment of the respective species (19–22). Despite the convenience of raising mantises in terrariums, they can pose a number of challenges such as limited space, irregular feeding times and cannibalism. Using an automatic feeder has recently been found to alleviate these limitations by providing a consistent and individualized feeding mechanism for each mantis (23). Besides food availability, water and a constant temperature and relative humidity (RH) regime to meet the species-specific requirements of mantises are crucial factors for optimal activity, metabolism, digestion, molting, mating and oviposition (7, 24–26). Exceptionally, some species from hot and arid regions thrive at temperatures of 35°C and higher while most species prefer daytime temperatures of 28–30°C. Temperate species like *M. religiosa* and *Tenodera sinensis* (Mantodea: Mantidae; Saussure, 1871) do better at 24–28°C. Proper ventilation is also a crucial factor as excessive humidity and poor air circulation has been shown to cause diseases (10). In terraristics, some of the most commonly kept species include the giant Asian mantis *Hierodula patellifera* (Mantodea: Mantidae; Serville, 1839) and the Ghana flower mantis *Chlidonoptera lestoni* (Mantodea: Hymenopodidae; Roy & Leston, 1975) due to their attractive appearance, relatively easy care and captivating hunting behavior (27, 28). *C. lestoni* originates from the western part of Africa, especially Ghana. Studies indicate that they are predatory generalists and consequently play an important role in regulating and maintaining biodiversity by controlling local pest populations (28). In comparison, *H. patellifera* is much larger and shows evidence of a more aggressive predatory behavior (29–31). It is native to parts of Asia, including China, Korea and Japan (32–34). Both species are very versatile in nature and demonstrate the ability to adapt easily to different ecological environments (27, 28).

In terraristics and research, different Diptera species are the most commonly used prey, along with crickets and insects of the order Blattodea (*Blaptica dubia* (Blaberidae; Serville, 1838), *Periplaneta lateralis* (Walker, 1868) and *P. americana* (Linnaeus, 1758; both Blattidae) amongst many others). Occasionally, lepidopterans such as *Galleria mellonella* (Linnaeus, 1758) and *Plodia interpunctella* (Hübner, 1813; both Pyralidae), coleopterans such as *Tenebrio molitor* (Linnaeus, 1758), *Zophobas morio* (Fabricius, 1776) and *Alphitobius diaperinus* (Panzer, 1797; all Tenebrionidae), as well as springtails and aphids are used. These may be sourced from the wild, grown at home or purchased from specialist producers (23). The choice of fly species is contingent upon the developmental stage and the corresponding size of the mantises. Hatchlings are typically nourished with fruit flies (*Drosophila melanogaster* (Meigen, 1830) and *D. hydei* (Sturtevant, 1921); Diptera: Drosophilidae), while growing and (sub)adult specimens are fed houseflies (*Musca domestica*; Diptera: Muscidae; Linnaeus, 1758), flesh flies (*Sarcophaga* sp. (Diptera: Sarcophagidae; Meigen, 1826), or blow flies, namely *Lucilia sericata* (Meigen, 1826) and *Calliphora* sp. (Diptera: Calliphoridae; Robineau-Desvoidy, 1830) (35). Given the praying mantis’ elevated demand for proteins, fats and other macronutrients, there is considerable interest in investigating alternative prey, such as the black soldier fly, *Hermetia illucens* (BSF; Diptera: Stratiomyidae; Linnaeus, 1758) (36). BSF larvae have attracted attention due to their remarkable ability to convert various organic side streams into protein- and fat-rich biomass (37), while their adults are mostly overlooked which is why data on their nutritional composition are scarce (38, 39). Despite their favorable size and simple rearing, BSF adults have not yet been considered for feeding praying mantises. Their natural behavior also makes them potentially attractive to mantises, which hunt in response to visual stimuli and movement patterns (10, 18). As a consequence, further research is essential to understand the dietary compatibility of BSF adults in different praying mantises and to identify the potential nutritional benefits.

Considering the increasing industrialization and availability of BSFs, the general goal of this pilot study was to evaluate the usability of BSF adults in feeding praying mantises. Based on randomized controlled feeding trials, we specifically:

1. analyzed the developmental progress and longevity of *C. lestoni* and *H. patellifera* fed with BSF adults,
2. observed and documented the acceptance and feeding behavior of both mantises, and
3. assessed the nutritional profiles of three commonly used feeding flies – *D. melanogaster*, *M. domestica* and *L. sericata* – and compared them with adult BSFs.

## 2 Material and methods

The experiments were conducted at the Fraunhofer Institute for Molecular Biology and Applied Ecology (Giessen, Germany) between March and August 2024.

### 2.1 Rearing and maintenance of insects

BSFs were provided by Illucens GmbH (Ahaus-Alstätte, Germany). There, after hatching from the eggs, the neonates were transferred to boxes measuring 52 × 32 × 12 cm (l × w × h) for 5 days and then fattened in trays measuring 600 × 50 × 20 cm (l × w × h) for a further 15 days under controlled conditions of 28 ± 2°C and 62 ± 7% relative humidity in darkness. The larvae were fed a high-quality diet consisting of rye meal and wheat bran. Once the majority of individuals had reached the prepupal stage, they were separated from the remaining substrate by sieving and sent to the Fraunhofer Institute. After receiving the package, the prepupae were immediately unpacked, transferred to 30 × 20 × 10 cm polypropylene boxes (l × w × h) and incubated in a climate chamber at 27 ± 1°C, 65 ± 5% relative humidity and constant darkness for pupation and metamorphosis. Adult flies were collected daily using spring steel tweezers and transferred into mesh cages (Bioform, Nuremberg, Germany), measuring 60 × 60 × 90 cm (l × w × h), located in the greenhouse at 26 ± 1°C, 60 ± 5% relative humidity, and with a 12 h photoperiod (SON-K 400 high-pressure sodium-vapor lamp, DH Licht, Wülfrath, Germany). Water was provided *ad libitum* using water-soaked paper towels. In addition, the mesh cages were sprayed daily with water (38).

*H. patellifera* and *C. lestoni* were obtained from Zoo & Co. (Hanau, Germany) and a private breeder (Butzbach, Germany), respectively. To avoid cannibalism, both species were kept individually in conical 8.4 × 8.4 × 11.4 cm (l × w × h) polypropylene boxes, which contained hazelnut branches for climbing and paper towels on the ground to soak up liquids. The lid was fitted with a circular 4.5 cm mesh insert, enabling gas exchange and water supply (39). Young praying mantises were fed *ad libitum* with *D. melanogaster* (L1–L3), *M. domestica* and *L. sericata* (≥L4) flies (Fauna Topics, Marbach am Neckar, Germany) every two days until they reached a size that allowed them to catch adult BSF. The boxes were thoroughly cleaned of uneaten fly(parts), excrements and molting residues once a week and a fresh paper towel was placed on the ground. The mantises were maintained at 25 ± 2°C, 55 ± 5% relative humidity and natural sunlight.

### 2.2 Feeding experiments

The feeding study was planned for a period of 90 days. Both species of praying mantis (both *n* = 6) were kept under the conditions described in section 2.1. Water was provided at two-day intervals by spraying ~2 mL of tap water, meticulously targeting the sides of the boxes, in a way that avoids harming the insects with the spraying pressure. The mantises were fed one or two flies every four days, depending on their appetite. For this purpose, flies that had emerged from the puparium no more than 48 h ago were caught from the mesh cages using spring steel tweezers. Flies of both sexes were randomized and fed to the mantises. The mantises were not disturbed during molting and were left untouched and unfed 24 h afterwards. In contrast to the young mantises (section 2.1), the boxes were thoroughly cleaned of uneaten fly(parts), excrements and molting residues every two weeks and a fresh paper towel was placed on the ground. Feeding behavior and time-dependent parameters, including longevity, frequency and time of molting, were documented daily.

### 2.3 Nutritional analysis of flies

All parameters were recorded as biological triplicates. Besides the moisture content, crude fat and protein data studied refer to the dry matter (DM) content. Frozen samples of *D. melanogaster*, *M. domestica* and *L. sericata* adults (maximum 48 h after emergence) were obtained from Fauna Topics (Marbach am Neckar, Germany) and first ground in a mortar under liquid nitrogen and subsequently lyophilized for 72 hours (Delta LSCplus, Martin Christ Gefriertrocknungsanlagen, Osterode am Harz, Germany). Before lyophilization, the moisture content was determined thermogravimetrically (M35 moisture analyzer, Sartorius, Göttingen, Germany). 0.5 g of lyophilized flies were used for total nitrogen and fat analyses. Total nitrogen and fat content were determined according to the Kjeldahl and Weibull-Stoldt methods, respectively, as described elsewhere (40). A conversion factor of 6.25 was used to calculate the crude protein content.

### 2.4 Data processing and statistics

Statistical analysis and visualization were carried out using Excel 2016 (Microsoft, Redmond, WA, USA) and OriginPro 2023b (OriginLab, Northampton, MA, USA). The homogeneity of variance was calculated with Levene’s test. Lifetime data were assessed using the non-parametric Kaplan–Meier estimator and the *S(t)* survival functions were compared pairwise using log-rank tests (α = 0.05). Significant differences in all other parameters related to the mantises were tested using Student’s or Welch’s t-test (41), depending on the homogeneity of variance with significance at 0.05. The chemical composition of flies was subjected to a one-way analysis of variance (ANOVA) and mean values were separated using Tukey’s test (homogeneous variance). If the assumption of homogeneity of variance was not met, Welch’s one-way ANOVA followed by a Games-Howell *post hoc* test was performed (42).

## 3 Results

### 3.1 Molting and longevity

**Figure 1:**
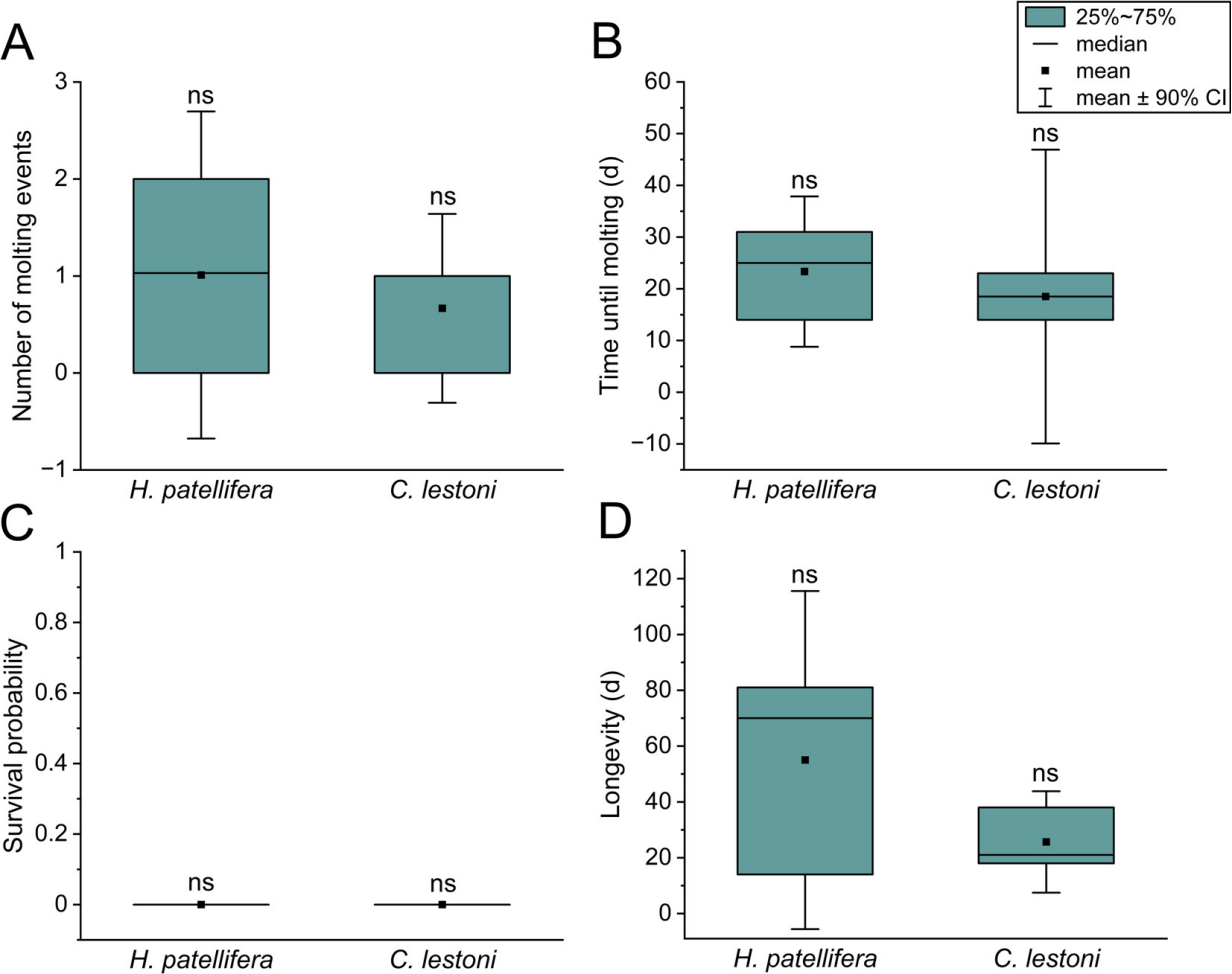
Developmental and survival parameters of *H*. *patellifera* and *C. lestoni* fed with BSF adults. **(A)** Individual number of molting events and **(B)** time until molting. **(C)** survival probability and **(D)** longevity of mantises. Data are displayed as box-and-whisker plots (CI = confidence interval), with “ns” indicating no significant differences were found between the mantises (*p* < 0.05; Student’s or Welch’s t-test; Kaplan–Meier estimator for longevity-related data).

The mean number of molting events per individual was 0.7 (*C. lestoni*) and 1.0 (*H. patellifera*) and did not differ between the species (*p* = 0.64; **Figure 1A**). One specimen of each species did not molt. A second molting event could only be recorded in one representative of *H. patellifera*, which occurred 25 days after the first one. In general, all mantises that molted exhibited no complications during and after the process, and resumed feeding 1–2 days after their exoskeleton had solidified. The average time to molt did not differ significantly between species (*χ^2^* = 1.04; *p* = 0.31), but was 20.6% shorter in *C. lestoni* compared to *H. patellifera* (18.5 and 23.3 days; **Figure 1B**). The first molting event was observed 14 days after the start of feeding BSF adults to both species, whereas the last molting for *C. lestoni* and *H. patellifera* was documented after 23 and 39 days, respectively.

None of the test animals, both for *C. lestoni* and *H. patellifera*, survived the entire predetermined period of the feeding study (*χ^2^* = 1.18; *p* = 0.28; **Figure 1C**). Nevertheless, differences were found in terms of longevity. At 70 days, the LT_50_ (time until ≥50% died) was 3.3-fold longer in *H. patellifera* than in *C. lestoni*. Representatives of *H. patellifera* (55.0 days) lived 2.1-fold longer than those of *C. lestoni* (25.7 days), however, no significant difference was calculated (*p* = 0.25; **Figure 1D**). We recorded striking inter-individual variations in longevity within each species, with values ranging between 14–81 days and 18–38 days for *H. patellifera* and *C. lestoni*, respectively. Interestingly, the individuals with the shortest lifespan (≤18 days) were unable to molt, despite sufficient feed intake, which was indicated by a well-filled abdomen. At 53 days, post-molting longevity was 4.8-fold longer for *H. patellifera* compared to *C. lestoni*, suggesting a better adaptation to the BSF diet (*p* = 0.01). In general, longevity was positively correlated with time of molting (*r* = 0.76; *p* = 0.04).

### 3.2 Feeding behavior

Alongside longevity and developmental progress, feeding behavior and feed acceptance were also examined. In general, both *H. patellifera* and *C. lestoni* with a size of 2.0–2.5 cm accepted adult BSFs as prey (≥68% of body size) and were able to catch, hold and consume them using their raptorial prothoracic legs. Flies were usually ingested within 24 hours after being placed in the boxes, although occasionally individuals were consumed after two to three days.

After a prey item was introduced into the box, the mantises either showed no initial reaction or watched the new prey motionlessly. As the prey moved towards the mantis, it exhibited typical rocking behavior, characterized by rhythmic, repetitive sideways movements. BSF adults that came within the range of the forelegs were grabbed immediately. In rare cases, especially when BSF adults flew quickly towards the mantis, they were sometimes fended off using the forelegs and seized at a later time. Only living prey was caught and consumed directly. While both mantis species consumed *Drosophila*, *Musca* and *Lucilia* flies completely, except for individual limbs that fell off the body during ingestion, we observed selective feeding behavior when BSF adults were provided. The thorax and abdomen of BSF adults were usually opened ventrally or laterally using their mouthparts and the nutritious innards (fat body, immature eggs, etc.) were selectively consumed. Here, the feeding hole was usually located in the area of the translucent abdominal window of the flies. Interestingly, no dorsal feeding hole was recorded. The chitinous exoskeleton and its appendages (antennae, legs, and wings) were not eaten and were left on the bottom of the boxes. The head of the flies remained mostly intact. In total, only three individual flies were not eaten, but no catching behavior was observed. These were removed and inspected during the fortnightly cleaning of the boxes. Non-consumed BSF adults died of natural causes and were not injured or killed by the mantids. Both *H. patellifera* and *C. lestoni* had a well-fed abdomen at the time of death and were defecating regularly, indicating adequate food intake. However, the abdomen of the mantises exhibited broad, dark brown discolored segmental areas (3–5 mm) anteroventrally before death.

### 3.3 Nutritional composition of BSF adults

Since the nutritional composition of a prey can affect the development and survival of praying mantises, the BSF adults and conventionally used fly species were analyzed. The species studied are suitable for feeding different instars and showed pronounced differences in their weight, with BSF adults being ≥3.1-fold heavier than any of the others (*p* < 0.00001; **Table 1**). Furthermore, BSF adults had the highest dry matter content, followed by *M. domestica* (*p* < 0.0001). Total nitrogen and crude protein contents were lowest in BSF adults and *L. sericata* (*p* = 0.03 and *p* < 0.0007), although all values were very high at >51%DM. Contrastingly, crude fat content of BSF adults was highest (*p* < 0.00001), while that of *M. domestica* was up to 5.5-fold lower compared to the other species (*p* < 0.00001).

**Table 1:** Weight and chemical composition of fly species conventionally used for feeding praying mantises (*D. melanogaster*, *M. domestica*, *L. sericata*) compared to BSF adults.

	Sampling size	*D. melanogaster*	*M. domestica*	*L. sericata*	*H. illucens*
Individual weight (mg)	*n* = 450	3.37 ± 0.22^a^	8.45 ± 0.91^b^	32.14 ± 2.56^c^	99.56 ± 5.32^d^
Dry matter (%)	*n* = 3	28.55 ± 0.11^a^	32.55 ± 0.13^b^	25.28 ± 0.32^c^	40.47 ± 0.37^d^
Total nitrogen (%DM)	*n* = 3	9.25 ± 0.04^a^	9.52 ± 0.07^ac^	8.26 ± 0.04^b^	8.22 ± 0.24^ab^
Crude protein (%DM)	*n* = 3	57.79 ± 0.25^a^	59.49 ± 0.45^ac^	51.65 ± 0.22^b^	51.38 ± 1.51^ab^
Crude fat (%DM)	*n* = 3	18.19 ± 0.68^a^	5.26 ± 0.58^b^	17.65 ± 1.32^a^	28.77 ± 1.70^c^

Data are means ± SD. Besides the dry matter content, values are given as a percentage of dry mass (%DM). Different superscript letters (a–d) indicate statistically significant differences between species (*p* < 0.05; one-way ANOVA or Welch’s ANOVA).

## 4 Discussion

Praying mantises as insectivores rely on a well-balanced proportion of macronutrients such as proteins and fats for reproduction, growth and to fuel their energy needs. Protein in the praying mantis is essential for growth during molting stages, muscle development, reproduction and synthesis of new tissue, whereas fats mostly serve as a significant source of energy (43). While both proteins and fats are essential macronutrients in all species of mantises, their nutritional requirements might vary species- and sexspecific (10, 44).

### 4.1 Praying mantises and the use of BSF as feed

The black soldier fly (BSF) has recently gained attention by researchers due to its potential as a sustainable feed source characterized by a high protein and fat content. They are relatively easy and inexpensive to rear especially as the adult flies survive only on water and they do not bite or spread any zoonotic diseases to humans. The BSF has also shown its potential in addressing the feed demand of various animals (45–48). Furthermore, adult BSF are considerably larger compared to other flies, which may extend feeding intervals (**Table 1**). This makes them attractive as a potential feed source for enthusiasts who keep praying mantises and other insects as pets.

In this study, the praying mantis species *C. lestoni* and *H. patellifera* were solely fed with BSF adults. During this feeding experiment, the prey consumption, developmental progress and the survival of the different species was closely monitored. It was observed that both species died after consuming the BSF, although the acceptance of the flies was equally high. Interestingly, besides the species tested here, we fed one individual of *Hymenopus coronatus* (Mantodea: Hymenopodidae; Olivier, 1792) with BSF adults and found no adverse effects (data not shown). This indicates that BSF, despite its dense nutrient profile, may have certain attributes that make it unsuitable for some mantis species. Moreover, also sex-specific differences might play a role. It is therefore essential to identify the optimal prey for different sexes and species of mantises and to determine the appropriate level of macronutrient intake required to enhance survival and reproduction in captivity and to reduce mortality rates, which can be affected by dietary imbalances.

### 4.2 Crude fat and protein requirements of mantises

Recent studies have reported insects to be a rich source of proteins and fats, with protein content typically ranging from 40–70%DM (49). Research on adult female *Pseudomantis albofimbriata* (Mantodea: Mantidae; Stal, 1860) has demonstrated that their protein and fat content was highly dependent on the prey they were fed. In this study, mantises were fed locust diets of different composition: one high in protein and low in fat, and the other low in protein and high in fat. Here, the differing fat and protein levels significantly affected egg production in *P. albofimbriata.* The high protein diet (67% protein, 9.2% fat) resulted in a marked increase of egg mass (43). The above diet, with an approximate protein/fat ratio of 7.3, is relatively similar to the ratios of prey insects analyzed in our experiments, such as *M. domestica* (10.66), *D. melanogaster* (3.18) and *L. sericata* (2.93), which are commonly consumed by mantises and are characterized by relatively high protein and low fat content. The BSF, in contrast, exhibited a protein/fat ratio of 1.79, indicating that these flies contain a significantly higher amount of fat compared to the other prey usually provided to mantises (**Table 1**).

Fats acquired from the consumption of BSF adults could play an essential role in energy storage, when fed to *C. lestoni* and *H. patellifera*. However, excessive fat intake without an adequate amount of protein might lead to metabolic disturbances. Studies have shown that a high fat diet can be overwhelmingly disruptive to the digestive processes in insects which have been reported to excrete excess fat once their fat limit has been attained. The process of excretion is energetically demanding and may negatively impact energy reserve, overall fitness and survival of the insect (50). For instance, a high-fat diet significantly shortened the life span of *D. melanogaster* and drastically reduced its tolerance to extreme cold compared to its counterparts on a regular diet. Further investigations have confirmed that a high-fat diet can overwhelm lipid metabolism, leading to lipotoxicity, disrupting cell function, signaling cell death and causing necrosis in various tissues (51, 52). Although no specific studies have been conducted on the effects of a high-fat diet in mantises, mantises share similar metabolic traits with other insects. Therefore, it could be assumed that the necrotic areas observed in the abdomens of *C. lestoni* and *H. patellifera* before and after death were due to their inability to digest fats properly, causing severe digestive issues.

### 4.3 Diversity in prey consumption, microbial communities and selective feeding behavior

Most mantises require a variety of prey for consumption to ensure a balanced nutrient intake (11, 53). A diverse diet plays a key role in shaping the gut microbiome of the mantis, as the different insects consumed harbor distinct microbial communities. Research has shown that mantises have the capacity to retain certain microbes in their gut to promote the development of specific microbial communities that are essential in the digestion of dietary compounds (44). Exclusive reliance on a single food source such as the BSF could therefore compromise the diversity and functionality of the gut microbiome, leading to inadequate digestion and absorption of key macronutrients. Offering BSF in combination with other prey species to promote a diverse gut microbiota may be a more optimal approach.

The characterization of the BSF gut microbiome has proven to be very challenging as the microbial community is multifactorially regulated by parameters such as life stage, diet, and several environmental stimuli (40, 54). While it has been reported that the gut microbes of the BSF have antimicrobial properties enabling them to diminish undesirable pathogens, some studies suggest that they could harbor significant amounts of harmful pathogens (55). Such entomopathogens could have colonized the mantids’ gastrointestinal tract after consumption, penetrated the gut barrier and led to the death (dark brown coloration).

Selective feeding by mantises, as observed in our study, is a well-described behavior and may be a response to avoid potentially toxic or indigestible parts, such as the heads and legs, while ingesting nutritious parts of the BSF, such as the abdomen and thorax. Some mantises, including *Hierodula membranacea* (Burmeister, 1838) and *Sphodromantis viridis* (Forskål, 1775; both Mantodea: Mantidae), have evolved the ability to manage the passage of toxins from prey through their midgut without being absorbed and gradually excreted. Other species, such as the Chinese praying mantis, *T. sinensis*, which regularly feeds on the poisonous monarch butterfly, *Danaus plexippus* (Lepidoptera: Nymphalidae; Linnaeus, 1758), first punctures the caterpillar’s skin and discards the midgut, which is filled with toxic plant material (56). This is in contrast to our findings, since *C. lestoni* and *H. patellifera* ate the whole thoracic and abdominal innards without differentiating between different inner compartments of the BSF. As adult BSF no longer ingest solid food and did not consume toxic plant material during the larval stage, the selective feeding behavior is likely to reflect the mantis’ preference for nitrogen-rich tissues (57).

### 4.4 Conclusions and future directions

Mantises are commonly considered to be generalist predators and therefore their feeding habits are usually underestimated for standardization to optimize resources invested under artificial conditions. Instead, this study encourages a more careful assessment of the ecological demands of the different species, thus promoting a more thorough understanding of them in nature for their application under artificial conditions.

Although BSF appears to have considerable advantages as a potential food source for pet insects due to its high protein content, ease of rearing and sustainability, its microbial properties and high fat content may limit its suitability as a primary diet for *C. lestoni* and *H. patellifera*. The specific dietary requirements of mantises, including a balanced protein/fat ratio and a diet consisting of a variety of prey, suggest that certain insect species may provide a more suitable nutrient profile. However, our observations suggest that other mantises may perform better with adult BSF. Therefore, testing further mantis species could be an interesting approach for future feeding experiments.

While the BSF may not be the ideal primary diet for mantises, it can be strategically combined with other, leaner prey to offer a balanced nutritional profile without overwhelming the mantis with excess fats. In addition, different diets not only affect the chemical composition of BSF, but also the associated microbiota, which could have a positive effect on the digestibility in mantises. Particularly, agro-industrial by-products were shown to have a significant effect on the fat and protein content of BSF, offering the promising opportunity to adapt these to the specific needs of mantises. However, when feeding such diets, larvae and resulting flies won’t reach the size described in this study. Another approach would be to feed older flies as they only consume water after emergence and feed on the fat body accumulated during the larval stage, gradually reducing the fat content.

## Data Availability

The original contributions presented in the study are included in the article/supplementary material. Further inquiries can be directed to the corresponding author.
